# Common Bean (*Phaseolus vulgaris* L.) Accumulates Most *S*-Methylcysteine as Its γ-Glutamyl Dipeptide

**DOI:** 10.3390/plants8050126

**Published:** 2019-05-14

**Authors:** Elham Saboori-Robat, Jaya Joshi, Aga Pajak, Mahmood Solouki, Motahhareh Mohsenpour, Justin Renaud, Frédéric Marsolais

**Affiliations:** 1 Genomics and Biotechnology, London Research and Development Centre, Agriculture and Agri-Food Canada, London, ON N5V 4T3, Canada; saboorielham@gmail.com (E.S.-R.); jayajoshi20@gmail.com (J.J.); Aga.Pajak@canada.ca (A.P.); Justin.Renaud@canada.ca (J.R.); 2 Department of Plant Breeding and Biotechnology, Faculty of Agriculture, University of Zabol, Zabol 538-98615, Iran; mahmood.solouki@uoz.ac.ir; 3 Department of Biology, University of Western Ontario, London, ON N6A 3K7, Canada; 4 Horticultural Sciences Department, University of Florida, Gainesville, FL 32611, USA; 5 Agricultural Biotechnology Research Institute of Iran (ABRII), Agricultural Research Education and Extension Organization (AREEO), Karaj 31585-845, Iran; mthrhm@abrii.ac.ir

**Keywords:** *Phaseolus vulgaris*, common bean, *S*-methylcysteine, homoglutathione, phytochelatin synthase, cysteine, methionine

## Abstract

The common bean (*Phaseolus vulgaris*) constitutes an excellent source of vegetable dietary protein. However, there are sub-optimal levels of the essential amino acids, methionine and cysteine. On the other hand, *P. vulgaris* accumulates large amounts of the γ-glutamyl dipeptide of *S*-methylcysteine, and lower levels of free *S*-methylcysteine and *S*-methylhomoglutathione. Past results suggest two distinct metabolite pools. Free *S*-methylcysteine levels are high at the beginning of seed development and decline at mid-maturation, while there is a biphasic accumulation of γ-glutamyl-*S*-methylcysteine, at early cotyledon and maturation stages. A possible model involves the formation of *S*-methylcysteine by cysteine synthase from *O*-acetylserine and methanethiol, whereas the majority of γ-glutamyl-*S*-methylcysteine may arise from *S*-methylhomoglutathione. Metabolite profiling during development and in genotypes differing in total *S*-methylcysteine accumulation showed that γ-glutamyl-*S*-methylcysteine accounts for most of the total *S*-methylcysteine in mature seed. Profiling of transcripts for candidate biosynthetic genes indicated that *BSAS4;1* expression is correlated with both the developmental timing and levels of free *S*-methylcysteine accumulated, while homoglutathione synthetase (*hGS*) expression was correlated with the levels of γ-glutamyl-*S*-methylcysteine. Analysis of *S*-methylated phytochelatins by liquid chromatography and high resolution tandem mass spectrometry revealed only small amounts of homophytochelatin-2 with a single *S*-methylcysteine. The mitochondrial localization of phytochelatin synthase 2—predominant in seed, determined by confocal microscopy of a fusion with the yellow fluorescent protein—and its spatial separation from *S*-methylhomoglutathione may explain the lack of significant accumulation of *S*-methylated phytochelatins.

## 1. Introduction

The common bean (*Phaseolus vulgaris* L.) is an important source of protein, dietary fiber, complex carbohydrates, vitamins, minerals and phenolic compounds [1,2,3,4,5]. However, its nutritional quality is restricted by a low concentration of the essential sulfur amino acids, methionine and cysteine. A large number of studies have focused on improving sulfur-containing amino acids in crops by transgenic development [6,7,8,9,10], synthetic protein synthesis [11] or traditional breeding [12]. Major seed proteins present in the common bean, including the 7S globulin phaseolin and lectin phytohaemagglutinin, have a low concentration of methionine and cysteine. On the other hand, the common bean accumulates non-protein sulfur amino acid derivatives such as γ–glutamyl-*S*-methylcysteine [13,14], *S*-methylcysteine [15,16,17] and *S*-methylhomoglutathione [18].

Two genetically related lines of the common bean, SARC1 and SMARC1N-PN1 differ in their composition of major storage proteins [19]. SMARC1N-PN1 is deficient in phaseolin and major lectins, through introgressions from *Phaseolus coccineus* and Great Northern 1140, respectively. SARC1 contains the lectin arcelin-1 introduced from a wild accession. SMARC1N-PN1 has an increased concentration of methionine and cysteine, by 10 and 70%, respectively. This increase occurs largely at the expense of total *S*-methylcysteine measured after acid hydrolysis, which is reduced by 70% [20]. This non-protein amino acid cannot replace methionine or cysteine in the diet [21]. Shifting sulfur from the non-protein amino acid pool to methionine and cysteine could be an effective strategy for protein quality improvement.

**Figure 1:**
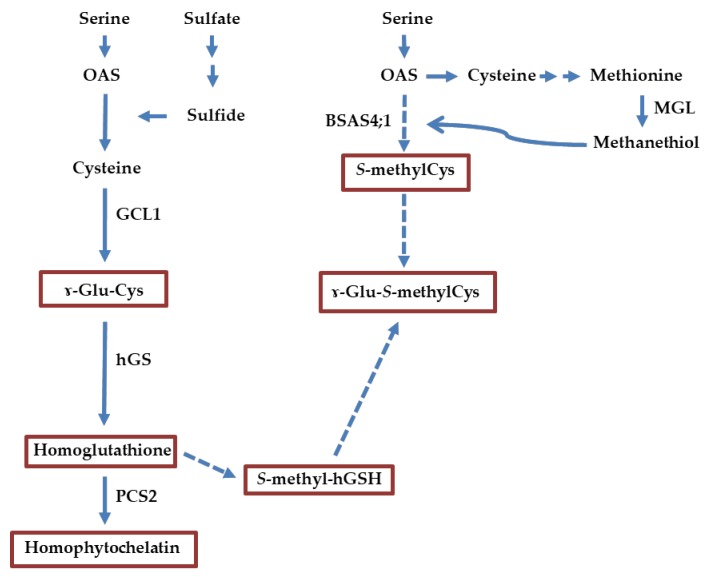
Possible biosynthetic pathway of sulfur amino acids in seed of the common bean. Solid arrows indicate steps taking place in seeds based on gene expression analysis. Multiple arrows refer to multiple steps. Broken arrows represent hypothetical steps. Metabolites in boxes are related to *S*-methylcysteine and were profiled in this study. The predominant pathway of cysteine biosynthesis in seed is cytosolic and *BSAS4;1* is the main cytosolic cysteine synthase expressed in seed. Formation of free *S*-methylcysteine by cysteine synthase is inferred from data in Arabidopsis. The presence of *S*-methylhomoglutathione suggests a pathway similar to that of the *S*-alk(enyl) sulfoxides in *Allium* leading to the formation of γ–glutamyl-*S*-methylcysteine (see text for details). OAS: *O*-acetylserine; γ–Glu-Cys, γ-glutamylcysteine; γ–Glu-*S*-methylCys, γ–glutamyl-*S*-methylcysteine; *S*-methyl-hGSH, *S*-methylhomoglutathione; BSAS4;1, β-substituted alanine synthase 4;1; PCS2, phytochelatin synthase 2; GCL1, glutamate–cysteine ligase 1; hGS, homoglutathione synthetase; MGL, methionine γ-lyase.

The biosynthesis of *S*-methylcysteine likely intersects with sulfur metabolism, particularly of cysteine. Sulfur is an essential macronutrient which plays a significant role in plant metabolism, increases yield and gives rise to the sulfur amino acids cysteine and methionine [22]. The most abundant environmental source of sulfur is sulfate (SO_4_^2-^). Utilizing this essential nutrient requires a series of steps for incorporation into metabolically active forms. This includes uptake of sulfate from the environment, reduction to sulfide and synthesis of cysteine [23,24,25,26]. Delivery of adequate sulfur to seed tissues is needed for maximizing yield and to improve protein quality [22]. During cysteine biosynthesis, several enzymes are active in developing seeds of the common bean (Figure 1) [18,27]. Serine acetyltransferase catalyzes the conversion of serine to *O*-acetylserine using acetyl-CoA [28,29]. Cysteine synthase is part of the β-substituted alanine synthase (BSAS) family, along with enzymes that use cysteine and cyanide to produce β-cyanoalanine [30]. One fate of cysteine is homoglutathione that is formed via a two-step process [31,32]. γ-Glutamylcysteine is synthesized from cysteine by glutamate–cysteine ligase, and homoglutathione from γ-glutamylcysteine by homoglutathione synthetase. Another fate of sulfur is to form homophytochelatins, through the reaction of phytochelatin synthase with homoglutathione as its substrate. Phytochelatins are cysteine-rich polypeptides that chelate toxic metals [33,34].

From past studies, a model emerges for possible pathways of *S*-methylcysteine biosynthesis. There appears to be two different metabolite pools, with a different pattern of temporal accumulation. Free *S*-methylcysteine concentration is relatively high at early developmental stages, and rapidly declines at mid-maturation, whereas γ–glutamyl-*S*-methylcysteine rapidly accumulates during the early cotyledon and maturation stages [35]. *BSAS4;1* represents the dominant cytosolic cysteine synthase expressed in developing seeds [36]. As can be inferred from data in Arabidopsis [37], this enzyme might be involved in the condensation of *O*-acetylserine and methanethiol, produced by methionine γ-lyase, to form *S*-methylcysteine (Figure 1). The presence of *S*-methylhomoglutathione suggests that a pathway similar to that of the alk(en)yl sulfoxides in *Allium* [38] might be involved in the formation of γ–glutamyl-*S*-methylcysteine in *P. vulgaris*. Since homoglutathione biosynthesis takes place in plastids, such a biosynthetic mechanism would ensure an absence of interference with the cytosolic pathway of cysteine biosynthesis, predominant in seed [39,40]. This hypothesis is consistent with the depletion of *O*-acetylserine in SMARC1N-PN1, associated with increased cysteine concentration [27].

In the present study, the different forms of *S*-methylcysteine and its possible precursors, including homoglutathione and *S*-methylhomoglutathione, were profiled during seed development and between genotypes that differ in total *S*-methylcysteine concentration. The relationship between metabolite levels and transcript levels of several candidate genes, including *BSAS4;1*, glutamate–cysteine ligase (*GCL1*), homoglutathione synthetase (*hGS*) and phytochelatin synthase (*PCS2*) was evaluated. The subcellular localization of PCS2 was determined and the possible presence of *S*-methylated phytochelatins analyzed by liquid chromatography and tandem mass spectrometry (MS/MS). The results of these experiments provide further insight into *S*-methylcysteine biosynthesis in *P. vulgaris*.

## 2. Results

### 2.1. Analysis of Sulfur Metabolite Profiles During Seed Development

To obtain more information on the accumulation of the different forms of *S*-methylcysteine and its precursors, free amino acids were profiled at seven developmental stages in BAT93 seed by high pressure liquid chromatography (HPLC) after derivatization with 3-mercaptopropionic acid and *O*-phthalaldehyde (Table 1). Developmental stages are designated after Walbot et al. [41]. Stages IV–early cotyledon to VI–early maturation are characterized by the presence of storage protein transcripts, while storage proteins accumulate from stages V–mid-cotyledon to VII–mid-maturation [42]. Stage VI–early maturation is marked by a decrease in photosynthetic capacity, with white cotyledons by stage VII–mid-maturation. Amino acids had been profiled previously using a different methodology involving derivatization with phenyl isothiocyanate [35]. This time, additional sulfur metabolites were quantified, namely homoglutathione and *S*-methylhomoglutathione, as possible precursors to γ-glutamyl-*S*-methylcysteine. Attempts to measure the homoglutathione precursor, γ-glutamyl-cysteine, were unsuccessful. Its levels were too low for accurate quantification using the present method. Accumulation of γ-glutamyl-*S*-methylcysteine followed a biphasic curve, as previously observed, with a steady rise from stage IV–early cotyledon until stage VI–early maturation, followed by a lag, and resumption of accumulation at stage VIII–late maturation. The levels of free *S*-methylcysteine were high at the first two stages of seed development, followed by a rapid decline during the late cotyledon and maturation stages. Concentrations of *S*-methylhomoglutathione were also higher at the beginning of seed development, when γ-glutamyl-*S*-methylcysteine accumulation is most rapid. Concentrations of homoglutathione were in the same range as those of *S*-methylhomoglutathione.

### 2.2. Differences in Sulfur Amino Acid Concentrations between SARC1 and SMARC1N-PN1 under Sulfate Sufficient Conditions

**Figure 2:**
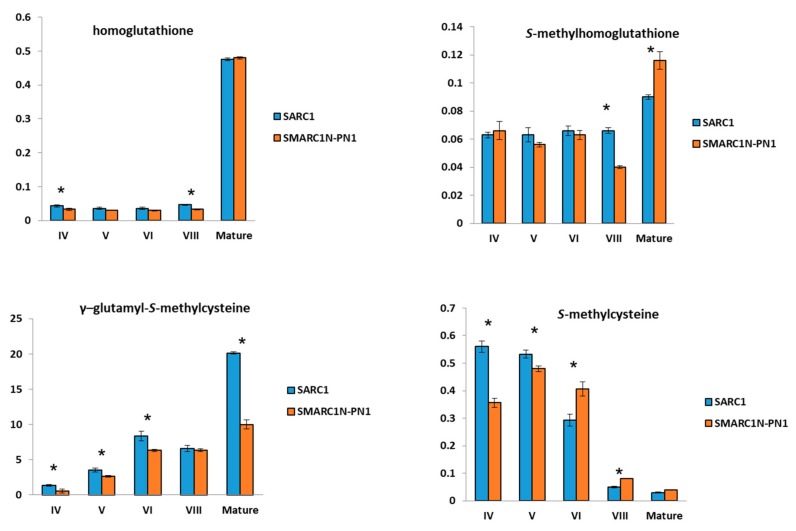
Quantification of sulfur metabolites at different developmental stages in SARC1 and SMARC1N-PN1. Concentration is expressed in nmol per mg seed weight; average ± standard deviation; *n* = 3. Asterisks indicate significant differences at *t*-test *p* value ≤ 0.01. *n* = 3.

The same sulfur metabolites were quantified at five developmental stages between SARC1 and SMARC1N-PN1 under defined, sulfate sufficient conditions. Free amino acids had been profiled before in SARC1 and SMARC1N-PN1, however, the levels of homoglutathione and *S*-methylhomoglutathione had not been determined [27]. The work by Pandurangan et al. [43] indicated that 2 mM is a suitable sulfate-sufficient concentration, at which the two genotypes accumulate different levels of total *S*-methylcysteine after hydrolysis. As expected, levels of γ-glutamyl-*S*-methylcysteine were higher in SARC1 than SMARC1N-PN1, at four out of five developmental stages (Figure 2). Similar to what was previously observed, the levels of free *S*-methylcysteine were higher in SARC1 than SMARC1N-PN1 at early developmental stages, when concentrations are most elevated. The concentration of *S*-methylhomoglutathione was higher in SMARC1N-PN1 at maturity, consistent with a reduced use of the putative γ-glutamyl-*S*-methylcysteine precursor in this genotype. Homoglutathione concentration was higher in SARC1 at stages IV and VIII, and *S*-methylhomoglutathione concentration at stage VIII, suggesting a possible enhanced flux through these putative precursors at these developmental stages.

### 2.3. Expression Analysis of Genes Related to S-Methylcysteine and γ-Glutamyl-S-methylcysteine Biosynthesis 

**Figure 3:**
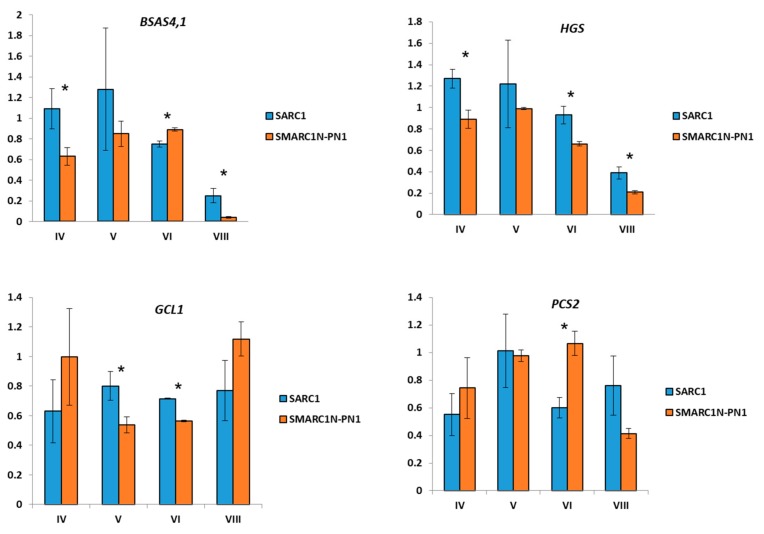
Relative expression of transcripts at different developmental stages in SARC1 and SMARC1N-PN1 determined by RT-qPCR. Average ± standard deviation. Asterisks indicate significant differences at t-test *p* value ≤ 0.02. *n* = 3.

The expression patterns of candidate genes related with the biosynthesis or metabolism of *S*-methylcysteine or γ-glutamyl-*S*-methylcysteine, *BSAS4;1*, *GCL1*, *hGS* and *PCS2*, were examined (Figure 3). *BSAS4;1* is the predominantly expressed cytosolic cysteine synthase gene in developing seeds [36]. There are two *GCL* genes in *P. vulgaris*. *GCL2* is expressed at very low levels, whereas *GCL1* and *hGS* expression is developmentally correlated (Pearson correlation coefficient = 0.85) (visualized at https://phytozome.jgi.doe.gov) [44]. Expression of the two glutathione synthetase (*GS*) genes is marginal, which explains the low level of accumulation of glutathione in *P. vulgaris*. Among two *PCS* genes, expression of *PCS1* was very low. Attempts to detect its expression by reverse transcription quantitative polymerase chain reaction (RT-qPCR) were unsuccessful. Expression of *BSAS4;1* was higher in SARC1 at the first developmental stage, which correlates with the higher levels of free *S*-methylcysteine (Figure 3). There was also a correlation between the levels of *BSAS4;1* transcripts and free *S*-methylcysteine during development (Pearson correlation coefficient = 0.83 in SARC1 and 0.96 in SMARC1N-PN1). Expression of *hGS* was significantly higher in SARC1 at three out of four developmental stages and *GCL1* at two developmental stages. This correlates with the higher levels of γ-glutamyl-*S*-methylcysteine in this genotype. *PCS2* transcript levels were higher in SMARC1N-PN1 at stage VI, opposite to what was observed for *GCL1* and *hGS*.

### 2.4. Subcellular Localization of PCS2

**Figure 4:**
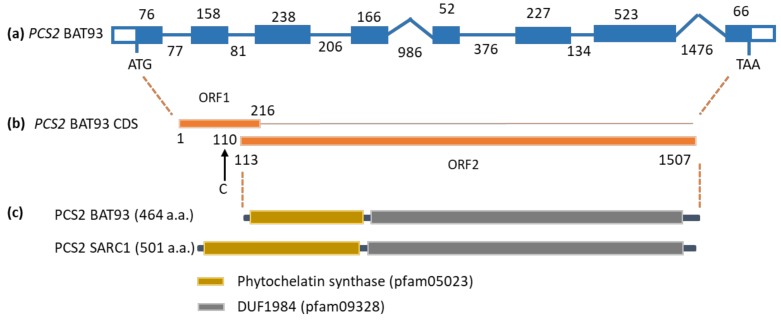
Naturally occurring variant of *PCS2* in BAT93. (**a**) Intron–exon structure of the BAT93 *PCS2* gene. The length of introns and exons is indicated starting from the first translation initiation codon and ending at the stop codon. (**b**) The gene gives rise to two open reading frames, due to the insertion of a cytosine at position 110 of the coding sequence (CDS), which results in a premature stop codon. Corresponding positions in the cDNA with respect to the first initiation codon is indicated. ORF1 encodes a predicted polypeptide of 71 amino acids. (**c**) Due to the insertion, *PCS2* encoded in BAT93 represents a shorter form translated from an alternative, downstream start codon as compared with *PCS2* encoded by SARC1, SMARC1N-PN1 and the reference bean genome, G19833. Pfam domains present in the phytochelatin synthase sequence are indicated.

*PCS2* is the major phytochelatin synthase gene expressed in developing seed. In vitro, *S*-methylglutathione can be used as substrate by phytochelatin synthase, in place of the metal thiolate complex with glutathione [45]. The subcellular localization of *PCS2* may therefore determine whether *S*-methylated phytochelatins can accumulate in seed. While analyzing the *PCS2* sequence from BAT93, a polymorphism specific to this genotype was uncovered which affects the length of the predicted PCS2 protein (Figure 4) [46]. This polymorphism was confirmed by RT-PCR and DNA sequencing. There is an insertion of a cytosine after position 109 downstream from the first start codon, as compared with the sequence from SARC1, SMARC1N-PN1 and the reference genome (accession G19833) [47]. The polymorphism results in a frameshift, such that PCS2 may only be translated from a downstream, alternative start codon, resulting in a shorter protein of 464 amino acid residues as compared with the longer PCS2 of 501 residues.

**Figure 5:**
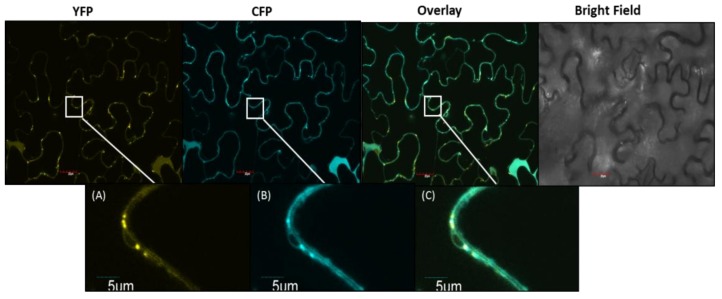
Subcellular localization of PCS2. (**a**) YFP-tagged PCS2; (**b**) CFP-tagged mitochondrial marker (Mt-CFP); (**c**) Co-localization of PCS2 with Mt-CFP. YFP: Yellow fluorescent protein; CFP: Cyan fluorescent protein.

cDNAs encoding the long and short versions of *PCS2* were cloned from SARC1 and constructs were made to express C-terminal yellow fluorescent protein (YFP) fusions transiently in *Nicotiana benthamiana* epidermal cells. Figure 5 shows representative results obtained with the longer version of the protein. When PCS2-YFP was co-expressed with a CFP-tagged mitochondrial marker protein, PCS2-YFP was co-localized with the marker to the mitochondria. Similar results were obtained with the shorter version of the protein.

### 2.5. Analysis of S-Methylated Phytochelatins

To determine whether *S*-methylated phytochelatins could constitute a significant sink for *S*-methylcysteine, a systematic analysis of mature seed extracts from BAT93, SARC1 and SMARC1N-PN1 was performed by liquid chromatography and MS/MS. Similar results were obtained for the three genotypes. There are 12 distinct phytochelatins and homophytochelatins with 2–7 repeat units. However, allowing for the possibility that phytochelatins and homophytochelatins may incorporate variable numbers of *S*-methylcysteines instead of cysteines increases this number to 28 possible phytochelatins, homophytochelatins and *S*-methylated analogues (Table S1). The full MS data was screened for the theoretical *m/z* of these compounds (< 3 ppm). When putatively detected, the profiles were scrutinized for the presence of the ^34^S isotope and MS/MS was performed.

**Figure 6:**
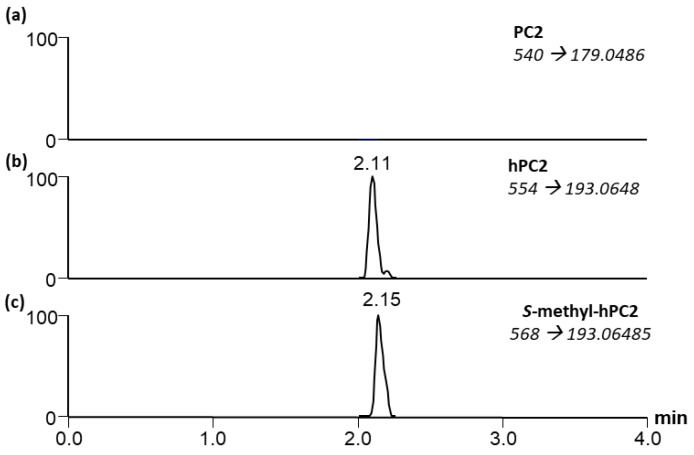
MS/MS chromatograms for monitoring (**a**) phytochelatin-2 (PC2); (**b**) homophytochelatin-2 (hPC2) and (**c**) *S*-methylhomophytochelatin-2 with a single *S*-methylcysteine (*S*-methyl-hPC2).

In these extracts, the most abundant compound based on relative peak areas was γ-glutamyl-*S*-methylcysteine, followed by homoglutathione and *S*-methylhomoglutathione. γ-Glutamylcysteine, *S*-methylglutathione and glutathione were present at lower concentrations. Homophytochelatin-2 and *S*-methylhomophytochelatin-2 with a single *S*-methylcysteine were detected (Figure 5). Their concentration was similar and in the same range as that of glutathione. Phytochelatin-2 was not detected (Figure 6a). A phytochelatin-2 that contains a single *S*-methylcysteine residue is isobaric with homophytochelatin-2 which contains an alanine residue in place of glycine and would not be distinguishable by high resolution MS. However, homophytochelatin and phytochelatin can be distinguished by their MS/MS product ions. Upon MS/MS, phytochelatins and homophytochelatins yield *m/z* 179.0486 and 193.0648 product ions, respectively. Within the analyzed samples, homophytochelatin-2 was detected and distinguished from *S*-methylphytochelatin-2 by observing this product ion (Figure 6b). Additionally, *S*-methylhomophytochelatin-2 with a single *S*-methyl group was detected with slightly more retention than homophytochelatin-2, as is expected by the additional methyl group (Figure 6c). Although a standard of *S*-methylhomophytochelatin-2 with two *S*-methyl cysteines was utilized, this compound was not detected in the seed extracts. No larger phytochelatin oligomers (<7) were detected within the samples by high resolution MS. Based on its low relative abundance, *S*-methylhomophytochelatin does not appear to constitute a major sink for *S*-methylcysteine.

## 3. Discussion

### 3.1. Most of the S-Methylcysteine Accumulates as γ-Glutamyl-S-methylcysteine in P. vulgaris Seed

The present study revisited the quantification of γ-glutamyl-*S*-methylcysteine in seed. Taylor et al. [20] reported that γ-glutamyl-*S*-methylcysteine accounted for only approximately 35% of total *S*-methylcysteine. Here, the final levels of γ-glutamyl-*S*-methylcysteine were about 3-fold higher than previously reported [14,35], in line with the concentration of total *S*-methylcysteine, measured after acid hydrolysis of mature seed flour, ranging from 14 to 35 nmol per mg seed weight [15,17,20]. We conclude that γ-glutamyl-*S*-methylcysteine accounts for most of the *S*-methylcysteine accumulated in mature seed. Giada et al. [14] determined that the average concentration of γ-glutamyl-*S*-methylcysteine was equal to 11 nmol per mg, with free *S*-methylcysteine accounting for the balance of the remaining 20% of total *S*-methylcysteine measured after acid hydrolysis. In the present study, a lower concentration of free *S*-methylcysteine was measured. The higher levels of free *S*-methylcysteine measured by Giada et al. [12] may have been due to partial hydrolysis of the dipeptide during extraction, or in vivo, such as during seed storage. The difference in γ-glutamyl-*S*-methylcysteine levels between BAT93 and SARC1 suggests that there may be substantial genetic variability for the concentration of this metabolite (Table 1 and Figure 2). In future, it may be useful to quantify γ-glutamyl-*S*-methylcysteine or total *S*-methylcysteine in a wide range of *P. vulgaris* accessions or cultivars.

### 3.2. The Concentration of Homoglutathione or S-Methylhomoglutathione does not Appear to Limit the Accumulation of γ-Glutamyl-S-Methylcysteine

*S*-Methylhomoglutathione concentration measured in mature seed of BAT93 in the present study was slightly higher than previously reported [18] and similar to that in *Vigna radiata* seeds [13]. Quantification of homoglutathione and *S*-methylhomoglutathione at different time points during seed development in SARC1 and SMARC1N-PN1 did not reveal major fluctuations with respect to the different levels of accumulation of the end-product, γ-glutamyl-*S*-methylcysteine (Figure 2). This is in contrast with the cysteine precursor, *O*-acetylserine, which was shown to be depleted in SMARC1N-PN1, in relation with the higher concentration of total cysteine in this genotype [27]. In BAT93, the decrease in *S*-methylhomoglutathione at early stages of seed development paralleled a rapid increase in γ-glutamyl-*S*-methylcysteine levels (Table 1).

### 3.3. Transcript Expression of BSAS4;1 and hGS is Correlated with the Accumulation of Free S-Methylcysteine and γ-Glutamyl-S-methylcysteine, Respectively

The product of *BSAS4;1* is presumed to be directly involved in the formation of free *S*-methylcysteine. The developmental correlation between *BSAS4*;*1* transcript levels and free *S*-methylcysteine concentration, observed in either SARC1 or SMARC1N-PN1 (Figure 2 and Figure 3), takes its meaning in this context. The positive genotypic correlation, observed at an early time point, when *S*-methylcysteine is at its highest levels, further implicates *BSAS4*;*1* as a plausible candidate gene (Figure 2 and Figure 3). The higher levels of *hGS* transcript in SARC1 as compared with SMARC1N-PN1 at three out of four developmental stages (Figure 3) supports the idea that flux through homoglutathione synthetase may control, at least in part, γ-glutamyl-*S*-methylcysteine accumulation.

### 3.4. Mitochondrial Localization of PCS2 Prevents the Accumulation of S-methylated Phytochelatins 

The presence of *S*-methylhomoglutathione throughout seed development raises the question of whether *S*-methylphytochelatins can accumulate in *P.* vulgaris. Analysis of *PCS2*, the major phytochelatin synthase gene expressed in seed, revealed a polymorphism in BAT93 which would result in alternative translation initiation at a downstream start codon (Figure 4). Determination of subcellular localization by confocal microscopy demonstrated that both variants are targeted to mitochondria (Figure 5). Information on the subcellular localization of plant phytochelatin synthases is relatively limited. Arabidopsis PCS1 was shown to be present in the cytosol [48], and rice PCS1 and PCS2 were also reported to be cytosolic [49]. In the yeast *Schizosaccharomyces pombe*, phytochelatin synthase is localized in mitochondria [50]. This is logical, given that heavy metal toxicity targets mitochondrial respiration [51]. A systematic analysis of *S*-methylated phytochelatins in seed extracts found *S*-methylhomophytochelatin with a single *S*-methylcysteine, at low levels (Figure 6 and Table S1). The present results suggest that the localization of PCS2 in mitochondria ensures that the formation of phytochelatins does not interfere with the accumulation of γ-glutamyl-*S*-methycysteine.

In conclusion, our results indicate that most of the *S*-methylcysteine accumulates as γ-glutamyl-*S*-methylcysteine in mature seed. Analysis of metabolite profiles and quantitative RT-PCR data for transcripts of candidate genes of *S*-methylcysteine metabolism supports the hypothesis that expression of *BSAS4;1*, encoding the major cytosolic synthase in seed, may regulate the accumulation of free *S*-methylcysteine, whereas expression of *hGS* may regulate the accumulation of γ-glutamyl-*S*-methylcysteine, through the provision of homoglutathione and *S*-methylhomoglutathione. The mitochondrial localization of PCS2, the major phytochelatin synthase expressed in seed, is a likely explanation for the lack of substantial accumulation of *S*-methylated homophytochelatins.

## 4. Materials and Methods 

### 4.1. Plant Materials and Growth Conditions

Three genotypes of the common bean (*Phaseolus vulgaris* L.), SARC1, SMARC1N-PN1 and BAT93 were used to evaluate sulfur metabolite profiles. Seeds were sown in small trays containing vermiculite. Ten-day-old seedlings were transplanted to a bigger pot measuring 17 cm × 20 cm containing sand, perlite, and vermiculite in a 2:1:1 ratio. SARC1 and SMARC1N-PN1 plants were grown under sulfate sufficient conditions as described in previous works [43,52]. The sulfate solution included 0.2 mM K_2_SO_4_ and 1.8 mM MgSO_4_ with other nutrients as follows: 4.5 mM Ca(NO_3_)_2_, 1.7 mM K_2_HPO_4_, 4 μM MnSO_4_.H_2_O, 5 μM H_3_BO_3_, 10 μM FeEDTA, 0.25 μM CuSO_4_.5H_2_O, 1 μM ZnSO_4_.7H_2_O, and 0.2 μM Na_2_MoO_4_.2H_2_O. The BAT93 genotype was grown as previously described [35]. The pots used in the study were placed in a randomized block design with 15 plants per genotype. Each sample consisted of three biological replicates. A biological replicate consisted of a pool of 10 seeds collected randomly from 15 plants per genotype. Plants were grown in a greenhouse with 16 h light (300–400 μmol photons m^−2^ s^−1^) and 8 h dark with a temperature cycling between 18 and 24 °C.

### 4.2. Extraction and Quantification of Free Amino Acids

The frozen seeds in liquid nitrogen were ground to a fine powder using a mortar and pestle. Replicate samples consisted of independent pools of ten seeds of which 100 mg were used for extraction. Sample extraction was carried out in ethanol/water (70:30), optimal for sulfur containing γ-glutamyl dipeptides [20]. Standards for γ-glutamylcysteine, γ-glutamyl-*S*-methylcysteine, *S*-methylhomoglutathione, homoglutathione and *S*-methylhomophytochelatin-2 with two *S*-methylcysteines were from Bachem (Torrance, CA, USA). Homophytochelatin-2 and phytochelatin-3 standards were from Anaspec (Fremont, CA, USA). *S*-Methylglutathione was from Millipore Sigma (Oakville, ON, Canada). Quantification of free amino acids was performed after derivatization with *O*-phthalaldehyde and mercaptopropionic acid using an Agilent 1260 Infinity HPLC system (Mississauga, ON, Canada) as described in Jafari et al. [53].

### 4.3. RNA Extraction and Quantitative RT-PCR

Sequence analyses were performed using *Phaseolus vulgaris* v2.1, DOE-JGI and USDA-NIFA (http://phytozome.jgi.doe.gov/). Total RNA from the developmental seed samples was extracted using a modified lithium chloride method [54]. RNA was quantified with a NanoDrop 1000 (Thermo Fisher Scientific, https://www.thermofisher.com) and its quality evaluated from A_260_/A_280_ ratio. DNase I was used to remove DNA contamination that may have happened during the RNA extraction (Thermo Fisher Scientific). RNA quality was evaluated prior to cDNA synthesis by using gel electrophoresis on a 1% (w/v) agarose gel. cDNA synthesis and PCR procedures were performed using Thermoscript RT-PCR System (Thermo Fisher Scientific) and SsoFast EvaGreen Supermix (Bio-Rad Laboratories, Mississauga, ON, Canada), respectively. Primers used for RT-qPCR are listed in Table 2. Reactions contained 2 µL of cDNA diluted 5-fold, primers at a concentration of 0.5 µM and 5 µL SsoFast EvaGreen Supermix in a final volume of 10 µL. Samples were run in Hard-Shell 96-well clear PCR plates (Bio-Rad Laboratories). In each plate, three biological replicates, with three technical replicates per biological replicate and controls without template were run for developmental seed samples. The PCR program consisted in an initial step of 2 min at 95 °C, followed by 35 cycles of 30 s at 94 °C and 30 s at 60 °C. CFX Maestro software was used to analyze the RT-qPCR data. After completion of the reactions, the threshold fluorescence (C_q_) value for each reaction was calculated. All data were normalized using the expression of the ubiquitin reference gene. The specificity of primer pairs was confirmed by melt curve analysis in comparison with controls without template. PCR efficiency was calculated from a standard curve of C_q_ versus the logarithm of starting template quantity. Each assay was optimized so that the efficiency ranged between 98% and 108%, with a coefficient of determination (R^2^) > 0.98.

### 4.4. Cloning of PCS2 for Subcellular Localization 

The coding sequence of *PCS2* was PCR amplified using attB1 and attB2 site-containing Gateway primers from SARC1 cDNA. For the full-length version, primers were, PVPCS2-ATTB1YFPF, 5’-GGGGACAAGTTTGTACAAAAAAGCAGGCTTCATGTGCATGGCGAACCCAG-3’ and PVPCS2-ATTB1YFPR, 5’-GGGGACCACTTTGTACAAGAAAGCTGGGTTCCGCTGCACAGTCCAGATTGCT-3’. For the short variant using the alternative downstream start codon, the forward primer was PCS2-ATTB1YFPF, 5’-GGGGACAAGTTTGTACAAAAAAGCAGGCTTCATGGAAGCCTTCTTCAAGC-3’. The PCR products were inserted into the entry vector pDONR-Zeo (Thermo Fisher Scientific). The integrity of the PCR fragments was confirmed with Sanger sequencing. Using Gateway technology, LR recombination reactions were performed with entry clones pDONR-Zeo-PCS2fl and pDONR-Zeo-PCS2tr and destination vector pEarleyGate101 [55]. The final expression constructs (pEG101-PCS2fl and pEG101-PCS2tr) were transformed into *Agrobacterium tumefaciens* strain GV3101.

### 4.5. Plant Transformation and Confocal Observations

*Nicotiana benthamiana* plants were grown for 6 weeks and used for transient expression. Plants were grown in a growth chamber at 22 °C with a 16 h photoperiod (110 µmol photons m^−2^ s^−1^). Plants were always watered with the water soluble fertilizer (N:P:K = 20:8:20) at 0.25 g/L (Plant Products, Brampton, ON, Canada). *Agrobacterium tumefaciens* cultures were grown to an optical density at 600 nm (OD_600_) of 0.5–0.8, and collected by centrifugation at 3,000 × *g* for 30 min. The pellets were resuspended in Gamborg’s solution (3.2 g/L Gamborg’s B5 medium and vitamins, 20 g/L sucrose, 10 mM 2-(*N*-morpholino)ethanesulfonic acid pH 5.6, 200 µM acetosyringone) to a final OD_600_ of 1, followed by incubation at room temperature (21 °C) with gentle agitation for 1 h. For co-infiltration, the mitochondrial protein (cytochrome oxidase with CFP) was mixed with YFP construct (pEG101-PCS2) in a 1:1 ratio [56]. The suspension was used for co-infiltration of the abaxial side of the leaf with a 1 mL syringe. Three to four days post infiltration, the abaxial epidermis of the leaves was observed using an OLYMPUS FV1200 Laser Scanning Microscope (https://www.olympus-lifescience.com/). A 60 × water immersion objective was used at excitation wavelengths of 514 and 458 nm. The fluorescence signals were detected using an emission spectra of 530–560 nm for YFP and 470-500 nm for CFP. Sequential Scan Tool, which records fluorescence in a sequential fashion, was used for studying co-localization of PCS2 with marker protein.

### 4.6. Analysis of S-methylated Phytochelatins

Extraction of phytochelatins from mature seed tissue was performed according to a published method with slight modifications [57]. The mature seed samples were ground with a Kleco ball mill (Garcia Machine, Visalia, CA, USA). One hundred mg of powder was mixed with 1 mL of cold (4 °C) 100 mM dithiothreitol in a polypropylene centrifuge tube. The suspension was vortexed for 1 min, and then sonicated for 5 min at room temperature. The extract was precipitated by centrifugation at 15,000× *g* at 4 °C for 20 min. The supernatants were transferred to a polypropylene centrifuge tube and the centrifugation repeated one more time. The supernatants were filtered with PTFE syringe filters (0.22 µm) into 2 mL amber glass HPLC vials.

The extracts were screened using a Q-Exactive Quadrupole Orbitrap mass spectrometer (Thermo Fisher Scientific), coupled to an Agilent 1290 high-performance liquid chromatography (HPLC) system with a Zorbax Eclipse Plus RRHD C18 column maintained at 35 °C (2.1 × 50 mm, 1.8 µm; Agilent). Mobile phase A (0.1% formic acid in LC-MS grade H_2_O, Thermo Fisher Scientific) began at 100% and was held for 1.25 min. Mobile phase B (0.1% formic acid in LC-MS grade acetonitrile, Thermo Fisher Scientific) was then increased to 50% over 1.75 min, and 100% over 0.5 min. Mobile phase B was maintained at 100% for 1.5 min and returned to 0% over 0.5 min. The following heated electrospray ionization (HESI) parameters were used in positive ionization mode: spray voltage, 3.9 kV; capillary temperature, 250 °C; probe heater temperature, 450 °C; sheath gas, 30 arbitrary units; auxiliary gas, 8 arbitrary units; and S-Lens RF level, 60%. High resolution, full MS was used to detect any possible phytochelatins and homophytochelatins (PC2-7) by accurate mass (Table S1), while MS/MS scans performed concurrently monitored PC2 (*m/z* 540 → 179.0486), homoPC2 (*m/z* 554 → 193.0648) and *S*-methyl-homoPC2 (*m/z* 568 → 193.0648) all at normalized collision energies (NCE) of 24. The full MS scans were performed at 70,000 resolution over a mass range of 100–2000 *m/z*; automatic gain control (AGC) target and maximum injection time (max IT) was 3 × 106 and 256 msecond respectively. The MS/MS scans were performed at 17,500 resolution AGC target and max IT were 3 × 106 and 64 msecond respectively. Data were analyzed and all theoretical masses were calculated with Xcalibur™ software.

### 4.7. Statistical Analysis 

The experiments were carried out as a factorial in a completely randomized experimental design with three replications. *t*-Test was performed using IBM SPSS^®^ software (Armonk, NY, USA).

## Figures and Tables

**Table 1:** Free amino acid profiles in developing seeds of BAT93. Metabolites related to *S*-methylcysteine are highlighted.

Average Seed Weight (mg)/Develop-Mental Stage	25/IV- Cotyledon	52/V- Cotyledon	105/V-Cotyledon	157/VI- Maturation	200/VI- Maturation	348/VIII- Maturation	160/Mature
Asp	1.6 ± 0.1	0.80 ± 0.10	0.79 ± 0.02	1.2 ± 0.1	1.3 ± 0.1	1.4 ± 0.2	3.4 ± 0.4
Glu	4.5 ± 0.2	4.1 ± 0.2	4.0 ± 0.1	3.3 ± 0.1	2.9 ± 0.1	2.8 ± 0.1	4.4 ± 0.3
hGSH	0.06 ± 0.01	0.06 ±0.01	0.06 ± 0.01	0.12 ± 0.01	0.12 ± 0.02	0.11 ± 0.01	0.35 ± 0.06
Ser	1.3 ± 0.1	0.77 ± 0.09	0.56 ± 0.01	0.50 ± 0.01	0.39 ± 0.01	0.52 ± 0.12	0.24 ± 0.02
His	1.8 ± 0.1	1.3 ± 0.2	0.84 ± 0.02	0.98 ± 0.07	0.11 ± 0.01	0. 45 ± 0.29	2.5 ± 0.2
γ-Glu-***S***-methylCys	2.4 ± 0.1	7.0 ± 0.1	9.3 ± 0.2	10.2 ± 0.2	8.6 ± 0.3	11.4 ± 2.7	38.0 ± 2.0
Gly	0.33 ± 0.01	0.30 ± 0.01	0.25 ± 0.01	0.25 ± 0.01	0.24 ± 0.01	0.27 ± 0.03	0.58 ± 0.36
Thr	3.2 ± 0.1	3.3 ± 0.2	1.8 ± 0.1	1.6 ± 0.1	0.66 ± 0.02	1.3 ± 0.6	0.66 ± 0.06
***S***-methylhGSH	0.35 ± 0.02	0.28 ± 0.03	0.18 ± 0.01	0.17 ± 0.01	0.12 ± 0.01	0.13 ± 0.01	0.44 ± 0.04
Arg	5.2 ± 0.4	5.6 ± 0.4	2.9 ± 0.1	4.2 ± 3.2	0.41 ± 0.02	1.7 ± 1. 1	2.4 ± 0.2
Ala	2.6 ± 0.3	1.4 ± 0.1	1.3 ± 0. 1	0.82 ± 0.04	0.50 ± 0.02	0.44 ± 0.04	1.7± 0.1
γ-Glu-Leu	0.26 ± 0.05	0.39 ± 0.01	2.0 ± 0.1	1.3 ± 0.1	1.9 ± 0.1	1.1 ± 0.6	1.9 ± 1.4
Tyr	0.18 ± 0.03	0.19 ± 0.02	0.17 ± 0.01	0.13 ± 0.01	0.10 ± 0.01	0.08 ± 0.02	0.22 ± 0.02
***S***-methylCys	1.3 ± 0.1	1.3 ± 0. 1	0.43 ± 0.01	0.28 ± 0.01	0.13 ± 0.01	0.18 ± 0.05	0.32 ± 0.02
Val	1.8 ± 0.1	3.0 ± 0.3	0.94 ± 0.02	0.90 ± 0.05	0.41 ± 0.01	0.64 ± 0.21	0.93 ± 0.08
Met	2.9 ± 0.5	2.4 ± 0.1	0.75 ± 0.02	0.55 ± 0.03	0.24 ± 0.01	0.31 ± 0.07	0.38 ± 0.01
Trp	0.70 ± 0.09	0.38 ± 0.11	0.95 ± 0.03	0.66 ± 0.01	0.66 ± 0.01	0.51 ± 0.12	0.49 ± 0.01
Phe	0.47 ± 0.01	0.39 ± 0.05	0.20 ± 0.03	0.14 ± 0.01	0.09 ± 0.01	0.11 ± 0.02	0.37 ± 0.03
Ile	1.9 ± 0.1	3.7 ± 0.3	1.6 ± 0.1	0.90 ± 0.02	0.90 ± 0.01	0.77 ± 0.11	0.28 ± 0.04
Leu	4.0 ± 2.3	1.4 ± 0.5	2.2 ± 0.1	0.39 ± 0.38	0.36 ± 0.01	0.42 ± 0.06	0.26 ± 0.02
Lys	0.30 ± 0.02	0.59 ± 0.07	0.19 ± 0.01	0.27 ± 0.02	0.14 ± 0.07	0.19 ± 0.02	0.35 ± 0.03

Values presented are in nmol per mg seed weight; average ± standard deviation; *n* = 3. hGSH: homoglutathione; γ-Glu-*S*-methylCys: γ-glutamyl-*S*-methylcysteine; *S*-methylhGSH: *S*-methylhomoglutathione; *S*-methylCys: *S*-methylcysteine.

**Table 2:** Primer sequences used for RT-qPCR.

Gene	Accession Number	Forward Primer Sequence (5́–3́)	Reverse Primer Sequence (5́–3́)
*BSAS4;1*	Phvul.007G185200.2	GCGGCTGATGGTGGTTATATTT	CACCAGTTCCTATCCCTGCA
*GCL1*	Phvul.002G289200.1	TGCATTACCAGCACTTTGGG	ACATCTGTCTTTCTTCTGGGGT
*hGS*	Phvul.006G094500.1	CCGCAAAGAGAAGGAGGAGG	TGCTGGAAAAGTGGCTGGAA
*PCS2*	Phvul.001G162700.1	TTCTCTGGGAGGCAATGAGC	ACCTTCATCTCTACAACTCACAGT
*Ubiquitin*	Phvul.007G05260.1	ACAGCTGGAGGATGAAAGGA	GTCCGAACTCTCCACCTCAA

## Supplementary Materials

Table S1. Monitoring of phytochelatin ions in MS/MS data.
