# Supplementary material for: Common Bean (Phaseolus vulgaris L.) Accumulates Most S-Methylcysteine as Its γ-Glutamyl Dipeptide
Source: Plants (Basel). 2019 May 14;8(5):126. doi: 10.3390/plants8050126 (PMC6572574; doi:10.3390/plants8050126)
Supplement: Supplementary file 1 [file plants-08-00126-s001.pdf]

Table S1. Monitoring of phytochelatin ions in MS/MS data.

#SMC	Name	Sequence	Formula	m/z	Detected?
		**Phytochelatin (PC)**			
	PC2	γGlu-Cys-γGlu-Cys-Gly	C18H29N5O10S2	540.14286	N
	PC3	γGlu-Cys-γGlu-Cys-γGlu-Cys-Gly	C26H41N7O14S3	772.19464	N
	PC4	γGlu-Cys-γGlu-Cys-γGlu-Cys-γGlu-Cys-Gly	C34H53N9O18S4	1004.2464	N
	PC5	γGlu-Cys-γGlu-Cys-γGlu-Cys-γGlu-Cys-γGlu-Cys-Gly	C42H65N11O22S5	1236.2982	N
	PC6	γGlu-Cys-γGlu-Cys-γGlu-Cys-γGlu-Cys-γGlu-Cys-γGlu-Cys-Gly	C50H77N13O26S6	1468.35	N
	PC7	γGlu-Cys-γGlu-Cys-γGlu-Cys-γGlu-Cys-γGlu-Cys-γGlu-Cys-γGlu-Cys-Gly	C58H89N15O30S7	1700.4017	N
		**Homophytochelatin (hPC)**			
	hPC2	γGlu-Cys-γGlu-Cys-Ala	C19H31N5O10S2	554.15851	Y
	hPC3	γGlu-Cys-γGlu-Cys-γGlu-Cys-Ala	C27H43N7O14S3	786.21029	N
	hPC4	γGlu-Cys-γGlu-Cys-γGlu-Cys-γGlu-Cys-Ala	C35H55N9O18S4	1018.2621	N
	hPC5	γGlu-Cys-γGlu-Cys-γGlu-Cys-γGlu-Cys-γGlu-Cys-Ala	C43H67N11O22S5	1250.3138	N
	hPC6	γGlu-Cys-γGlu-Cys-γGlu-Cys-γGlu-Cys-γGlu-Cys-γGlu-Cys-Ala	C51H79N13O26S6	1482.3656	N
	hPC7	γGlu-Cys-γGlu-Cys-γGlu-Cys-γGlu-Cys-γGlu-Cys-γGlu-Cys-γGlu-Cys-Ala	C59H91N15O30S7	1714.4174	N
**#SMC**		***S*-methylCys PC and hPC analogues**			
1	SMC-GSH	γGlu-SMC-Gly	C11H19N3O6S	322.10673	Y
1	SMC-hGSH	γGlu-SMC-Ala	C12H21N3O6S	336.12238	Y
1	PC2	γGlu-SMC-γGlu-Cys-Gly	C19H31N5O10S2	554.15851	N
2	PC2	γGlu-SMC-γGlu-SMC-Gly	C20H33N5O10S2	568.17416	N
1	hPC2	γGlu-SMC-γGlu-Cys-Ala	C20H33N5O10S2	568.17416	Y
2	hPC2	γGlu-SMC-γGlu-SMC-Ala	C21H35N5O10S2	582.18981	N
1	PC3	γGlu-SMC-γGlu-Cys-γGlu-Cys-Gly	C27H43N7O14S3	786.21029	N
2	PC3	γGlu-SMC-γGlu-SMC-γGlu-Cys-Gly	C28H45N7O14S3	800.22594	N
3	PC3	γGlu-SMC-γGlu-SMC-γGlu-SMC-Gly	C29H47N7O14S3	814.24159	N
1	hPC3	γGlu-SMC-γGlu-Cys-γGlu-Cys-Ala	C28H45N7O14S3	800.22594	N
2	hPC3	γGlu-SMC-γGlu-SMC-γGlu-Cys-Ala	C29H47N7O14S3	814.24159	N
3	hPC3	γGlu-SMC-γGlu-SMC-γGlu-SMC-Ala	C31H49N7O14S3	828.25724	N
1	PC4	γGlu-SMC-γGlu-Cys-γGlu-Cys-γGlu-Cys-Gly	C35H55N9O18S4	1018.2621	N
2	PC4	γGlu-SMC-γGlu-SMC-γGlu-Cys-γGlu-Cys-Gly	C36H57N9O18S4	1032.2777	N
3	PC4	γGlu-SMC-γGlu-SMC-γGlu-SMC-γGlu-Cys-Gly	C37H59N9O18S4	1046.2934	N
4	PC4	γGlu-SMC-γGlu-SMC-γGlu-SMC-γGlu-SMC-Gly	C38H61N9O18S4	1060.309	N
1	hPC4	γGlu-SMC-γGlu-Cys-γGlu-Cys-γGlu-Cys-Ala	C36H57N9O18S4	1032.2777	N
2	hPC4	γGlu-SMC-γGlu-SMC-γGlu-Cys-γGlu-Cys-Ala	C37H59N9O18S4	1046.2934	N
3	hPC4	γGlu-SMC-γGlu-SMC-γGlu-SMC-γGlu-Cys-Ala	C38H61N9O18S4	1060.309	N
4	hPC4	γGlu-SMC-γGlu-SMC-γGlu-SMC-γGlu-SMC-Ala	C39H63N9O18S4	1074.3247	N
1	PC5	γGlu-SMC-γGlu-Cys-γGlu-Cys-γGlu-Cys-γGlu-Cys-Gly	C43H67N11O22S5	1250.3138	N
2	PC5	γGlu-SMC-γGlu-SMC-γGlu-Cys-γGlu-Cys-γGlu-Cys-Gly	C44H69N11O22S5	1264.3295	N
3	PC5	γGlu-SMC-γGlu-SMC-γGlu-SMC-γGlu-Cys-γGlu-Cys-Gly	C45H71N11O22S5	1278.3451	N
4	PC5	γGlu-SMC-γGlu-SMC-γGlu-SMC-γGlu-SMC-γGlu-Cys-Gly	C46H73N11O22S5	1292.3608	N
5	PC5	γGlu-SMC-γGlu-SMC-γGlu-SMC-γGlu-SMC-γGlu-SMC-Gly	C47H75N11O22S5	1306.3764	N
1	hPC5	γGlu-SMC-γGlu-Cys-γGlu-Cys-γGlu-Cys-γGlu-Cys-Ala	C44H69N11O22S5	1264.3295	N

2	hPC5	γGlu-SMC-γGlu-SMC-γGlu-Cys-γGlu-Cys-γGlu-Cys-Ala	$C45H71N11O22S5$	1278.3451	N
3	hPC5	γGlu-SMC-γGlu-SMC-γGlu-SMC-γGlu-Cys-γGlu-Cys-Ala	$C46H73N11O22S5$	1292.3608	N
4	hPC5	γGlu-SMC-γGlu-SMC-γGlu-SMC-γGlu-SMC-γGlu-Cys-Ala	$C47H75N11O22S5$	1306.3764	N
5	hPC5	γGlu-SMC-γGlu-SMC-γGlu-SMC-γGlu-SMC-γGlu-SMC-Ala	$C48H77N11O22S5$	1320.3921	N
